# Genome-scale DNA methylome and transcriptome profiling of human neutrophils

**DOI:** 10.1038/sdata.2016.19

**Published:** 2016-03-15

**Authors:** Aniruddha Chatterjee, Peter A. Stockwell, Euan J. Rodger, Ian M. Morison

**Affiliations:** 1 Department of Pathology, Dunedin School of Medicine, University of Otago, 270 Great King Street, Dunedin 9054, New Zealand; 2 Maurice Wilkins Centre for Molecular Biodiscovery, Level 2, 3A Symonds Street, Auckland 1010, New Zealand; 3 Department of Biochemistry, University of Otago, 710 Cumberland Street, Dunedin 9054, New Zealand

**Keywords:** Genetics research, DNA methylation, Epigenomics, RNA sequencing, Neutrophils

## Abstract

Methylation of DNA molecules is a key mechanism associated with human disease, altered gene expression and phenotype. Using reduced representation bisulphite sequencing (RRBS) technology we have analysed DNA methylation patterns in healthy individuals and identified genes showing significant inter-individual variation. Further, using whole genome transcriptome analysis (RNA-Seq) on the same individuals we showed a local and specific relationship of exon inclusion and variable DNA methylation pattern. For RRBS, 363 million, 100-bp reads were generated from 13 samples using Illumina GAII and HiSeq2000 platforms. Here we also present additional RRBS data for a female pair of monozygotic twins that was not described in our original publication. Further, We performed RNA-Seq on four of these individuals, generating 174 million, 51-bp high quality reads on an Illumina HiSeq2000 platform. The current data set could be exploited as a comprehensive resource for understanding the nature and mechanism of variable phenotypic traits and altered disease susceptibility due to variable DNA methylation and gene expression patterns in healthy individuals.

## Background & Summary

DNA methylation provides a stable epigenetic mechanism for the regulation of gene expression that is prevalent in all vertebrates^1^. Aberrant DNA methylation is a hallmark of several human diseases, including cancer^2^. In healthy individuals, variation in DNA methylation has been hypothesised to alter susceptibility to common diseases^3^ and response to drug treatments^4^. Firm evidence that variation in DNA methylation contributes to phenotypic variation and altered disease susceptibility is tantalising but scant^5^. In human cancer, aberrant methylation of promoters has been shown to silence genes; however, the relationship between DNA methylation changes and gene expression in normal individuals is less clear. In addition, the impact of variation of methylation outside promoter regions remains unknown.

Recently, we performed a large-scale genome-wide methylation analysis of neutrophils from 11 healthy individuals using reduced representation bisulphite sequencing (RRBS) technology^6^. Neutrophils were chosen as the most accessible homogeneous cell population. The assumption of our study was that early events that modify an individual’s methylation affect all cell types equally, as seen in mouse models^7^. In contrast if changes in DNA methylation can occur later in development they are likely to affect specific cell types (e.g., pancreatic islet cells) and in general these cannot be studied in humans. Our analysis identified extensive epigenetic variation in healthy individuals. Specifically, we were able to identify 12,851 distinct autosomal iVMF (inter-individual variably methylated fragments) and by overlaying chromatin state data we documented their association with genome regulation. Further, whole genome gene expression analysis on the same individuals showed a local and specific relationship of exon inclusion and variable methylation for the first time^8^.

**Figure 1:**
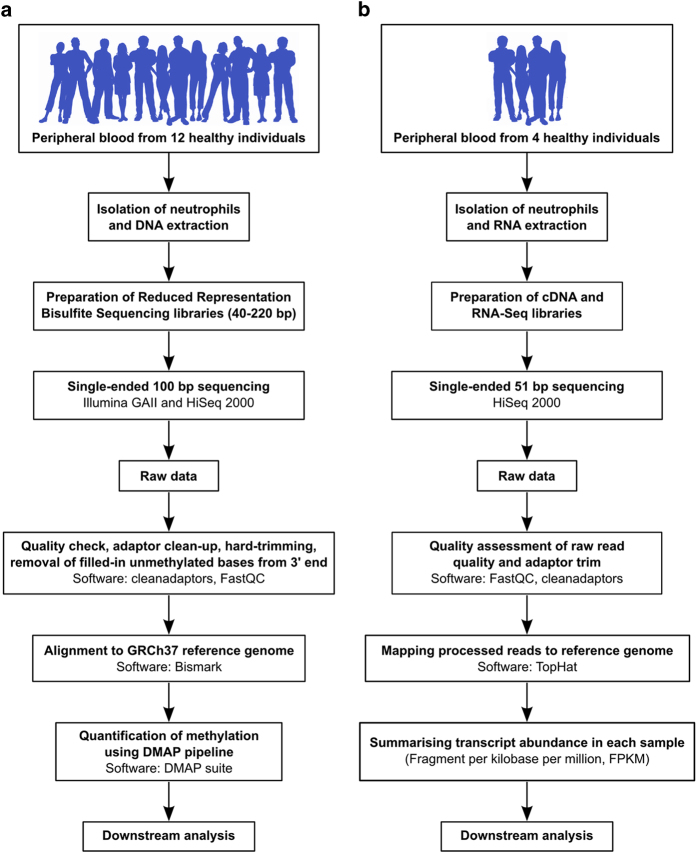
Description of the study and experimental and bioinformatics pipeline. (**a**) Overview of the main experimental and bioinformatic components and steps and used to generate genome-wide DNA methylation profiles from neutrophils of healthy individuals and identify inter-individual epigenetic variation. (**b**) Overview of the steps and approaches used to generate summaries of abundance of transcripts in healthy neutrophils and identify the relationship of exon usage and DNA methylation variation in our primary research paper.

In this work we present a total of 13 RRBS methylome and four transcriptome data sets for human neutrophils (Fig. 1a,b). RRBS provides base-resolution methylation information of four million CpG sites in the human genome (however, it doesn’t resolve 5-methylcytosine and 5-hydroxymethylcytosine^9,10^). For this study, peripheral blood was collected from healthy individuals of different ethnicity (age ranged from 25 to 34 years, median=31 years) and neutrophils were isolated (median purity=96%) for DNA extraction. For RRBS, we provide 12 data from samples that were described in our primary research (11 individuals and 1 technical replicate). In addition to that, in this article, we have added a new sample (X9017). Therefore, the RRBS data described here now contains one pair of female monozygotic twin samples (X9016 and X9017). Addition of monozygotic twin data provides opportunities for exploring the role of pure epigenetic variation^11^. In total, we generated 363 million, 100 bp, single-ended sequenced reads for RRBS experiments. Further, we also generated transcriptome profiles for four individuals (the same individuals as described in RRBS) using RNA-sequencing (RNA-Seq). For transcriptome profiles, we generated a total of 174 million, 51 bp, single-ended reads on the HiSeq platform. We describe a detailed workflow and analysis pipelines that will promote the use of this data and facilitate bioinformatic analysis of sequencing-based DNA methylation and transcriptome data in a broader context.

## Methods

We have presented some these methods and tools in our primary publication^8^ and previous publications^10,12^. This section integrates a collection of several methods that expand our previous descriptions to provide a comprehensive resource for reproducing both experimental and computational analysis.

### Description of ethical process

The goal and purpose of the project was described to each participant in detail. Informed consent was obtained from all subjects. Peripheral blood samples were collected from healthy individuals in accordance with the guidelines and approval obtained from the Dunedin Multi-region Ethics Committee, Dunedin, Otago region, New Zealand (approval number: MEC/09/07/068).

### Isolation of human neutrophils and extraction of DNA for RRBS

Neutrophils were extracted from 16 ml EDTA-anticoagulated blood that was diluted 1:1 in PBS and layered on Ficoll-Paque PLUS (GE Healthcare) and then centrifuged at 400×g for 40 min at room temperature (RT). The plasma and the ‘mononuclear’ layers were removed and discarded. To lyse the red blood cells, 0.17 M NH_4_Cl solution was added and incubated for 30 min at RT and then centrifuged at 300×g for 10 min. This lysis step was repeated. The neutrophil-rich pellet was resuspended in 2 ml PBS. The processed samples contained no dead cells, as assessed by trypan blue staining. Cell concentrations of the samples were determined using a Sysmex XE2100 haematology automated analyser. Cells were counted before and after neutrophil enrichment and only neutrophil enriched samples (>90%) were used for subsequent DNA extraction (Supplementary Table 1 and Supplementary Figs 1 and 2 and Supplementary File 1). DNA was extracted from the neutrophil suspension, using QIAamp DNA mini kit (Qiagen, Hilden, Germany) following the manufacturer’s protocol, with the modification that proteinase K treatment was performed overnight at 55 °C.

### RRBS library preparation

RRBS libraries were prepared according to our previously published protocols^10,13–15^. Briefly, genomic DNA was digested overnight with MspI (New England Biolabs, Ipswich, MA), followed by end-repair and addition of 3′-A overhangs. Methylated adaptors (Illumina, San Diego, CA) were ligated to the A-tailed DNA fragments. For reduced representation of the genome, 40 to 220 bp (pre-adaptor-ligation size) were excised from 3% Nusieve agarose gels (Lonza, Basel, Switzerland) after PCR. These fragments were bisulfite-converted with the EZ DNA methylation kit (Zymo Research, Irvine, CA). Bisulfite-converted libraries were amplified by PCR with 15–18 cycles (see gel image of three successful RRBS libraries in Supplementary Fig. 3, Supplementary File 1). Final RRBS libraries were quantified using the Qubit fluorometer (Life Technologies, Grand Island, NY). For quality assessment of the libraries, 1 μl of the final library was analysed on the 2100 Bioanalyzer (Agilent Technologies, Palo Alto, CA) using the high sensitivity DNA chip according to the manufacturer’s instructions.

### Sequencing of RRBS libraries, processing and alignment to reference genome

Two RRBS libraries (X9015 and X9006) were sequenced using the Illumina GAII platform and the other libraries were sequenced using the Illumina HiSeq2000 platform. A technical replicate of sample X9012 sample was also sequenced. The libraries were single-ended and the read length was 100 bp. Base-calling was performed by Illumina Real Time Analyzer (RTA) software; however, post-run standardization of the base-calling was performed using the Illumina Off-Line Base-calling application where applicable as previously described^13^.

**Figure 2:**
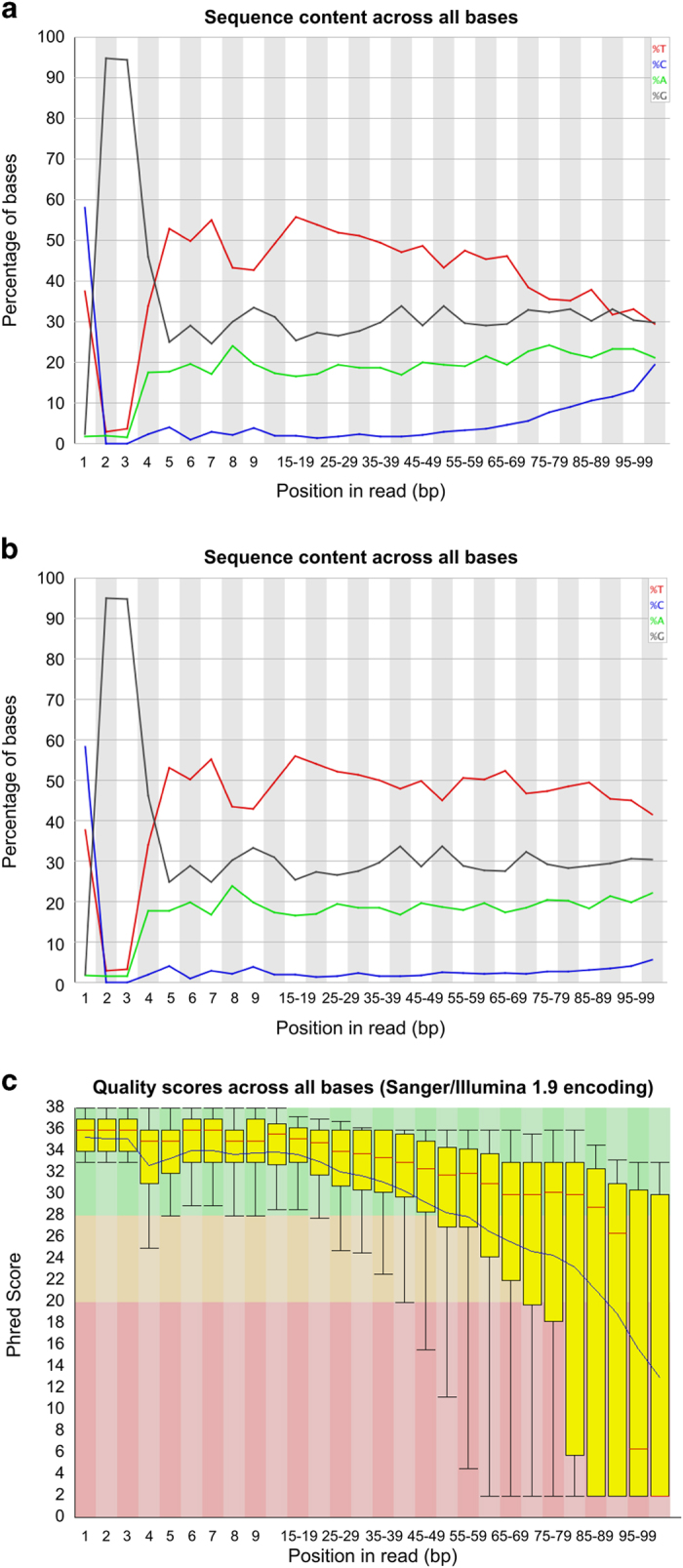
A representative example of signatures and quality of RRBS sequenced reads (sample: X9015). (**a**) Per base sequence content as indicated by FastQC of the raw sequenced reads. The X-axis plots the sequencing cycle or positions in reads. The Y-axis represents percentages if the occurrence of the bases along the read. (**b**) Per base sequence content as indicated by FastQC after adaptor sequence removal. (**c**) A representative example of per base sequence quality for RRBS data (sample: X9015). For each position a Box and Whisker plot of the Phred quality scores is drawn. The central red line is the median value. The yellow box represents the inter-quartile range (25–75%). The upper and lower whiskers represent the 10 and 90% points. The blue line represents the mean quality.

Quality of the sequenced reads for each individual sample was assessed using the FastQC program (see Code availability 1). FastQC data also confirmed the typical RRBS signature in the first three bases (at the 5′ end) of the reads (CGG or TGG sequences due to MspI digestion, see Figure 2a). Based on the quality, hard trimming of the sequence from 3′ end of the reads was performed with fastq_quality_trimmer v0.0.13 tool (see Code availability 2). Adaptor sequences were removed from the reads using our in-house *cleanadaptors* program^10^ (see Code availability 3). The *cleanadaptors* program scans the reads, identifying sections, which show 85% or higher matching with any of a series of adaptor sequences (the threshold is adjustable) in the sequenced reads and then the program removes the adaptor sequences from the sequenced reads. The output options include listing the source data with adaptor matches indicated in the listing (-f), or having the reads trimmed to remove any adaptor sequences that achieve criteria for matching (-F). Initially, the tool was developed for Illumina GAII sequencing operations but it was updated to be compatible with HiSeq outputs. The unmethylated CpG bases at the 3′ end of the reads were added during end-repair step and therefore, the sequence from the filled-in bases were removed using *cleanadaptors* (an optional switch (-t) is built into the program to remove these unmethylated filled-in bases). The sequenced reads were aligned against the complete human reference genome GRCh37 with the Bismark v0.6.4 aligner^16^ (see Code availability 4) with stringent criteria of one mismatch (default=2) in the seed (i.e., in the first 28 bp of the sequenced reads).

### Isolation of human neutrophils for transcriptome libraries:

For transcriptomics experiments, neutrophil RNA from four individuals was obtained. For each participant 20 ml of peripheral blood was collected into heparinized tubes. Enrichment for neutrophil was performed by a Dextran-Ficoll sedimentation and centrifugation method^17^. Briefly, 20 ml of peripheral blood was mixed with Dextran-RPMI media in a 4:1 ratio. After 40 min incubation at RT, the upper layer (white blood cell-rich plasma) was layered onto Ficoll (aspirate: Ficoll of 2:1 ratio) and centrifuged for 15 min at 2,500 rpm. To remove the remaining erythrocytes, the cell pellet was treated with 2 ml ddH_2_O for 10–15 s, after which 25 ml of RPMI was added. The suspension was centrifuged for 5 min at 1,400 rpm and the cell pellet was resuspended in 20 ml phenol red-free RPMI-1640. An aliquot of this suspension was used to check the presence of dead cells with trypan blue staining. This preparation contained >98% neutrophils. Total RNA was isolated using the RNeasy Mini Kit (Qiagen) following the manufacturer’s protocol. Two rounds of RNase-free DNase I digestion (Qiagen) was performed to eliminate genomic DNA during the extraction process. RNA concentrations were determined using a NanoDrop 2000 (Thermo Scientific, MA, USA). The quality and integrity of the RNA was determined using the RNA 6000 Pico chip on a 2100 Bioanalyzer (Agilent Technologies). The median RNA integrity number (RIN) for the 4 samples was 8.05. RNA libraries were constructed using 1 μg of total RNA with the TruSeq stranded mRNA Sample Preparation kit (Illumina) following the manufacturer’s protocol.

### Sequencing of RNA-Seq libraries, processing and alignment to reference genome

RNA was sequenced on the Illumina HiSeq 2000 sequencer (Illumina, USA) with a single-ended, 51-bp run producing raw fastq files. The quality of the RNA-Seq data was assessed using FASTQC program as described previously^18,19^. The RNA-seq reads were mapped to the human genome (assembly GRCh37) using TopHat (v2.0.11)^20^ (see Code availability 5), transcripts were assembled, abundances (Fragments Per Kilo base per Million or FPKM) of transcript were estimated. In our primary paper, for analysis of differential expression we used with Cufflinks (v2.2.0)^21^ package with default parameters. For exon usage analysis, Human gene models were flattened and reads assigned to exon bins and counted using HTSeq (v0.5.4p5)^22^. Differential exon usage was calculated using the DEXseq^23^ (v1.8.0) package.

### Annotation of genomic features

After determining the methylation status of the MspI fragments with high coverage, the next step was to annotate these fragments in respect to the genomic features. The *identgeneloc* program of the DMAP package (see Code availability 3) was used to associate fragments/ regions with their proximal genes and CpG features. *identgeneloc* is a command-line program which reads genomic feature table information and relates the MspI fragments (or any genomic region with a start and end) to annotated features. The application is capable of parsing feature table information from GenBank, EMBL, GTF, GFF3 and SeqMonk feature files, although it works optimally with the last of these. Gene annotations and CpG features were obtained from SeqMonk feature files (see Code availability 6). The SeqMonk tables are based on Ensembl annotation (see code availability 1). The SeqMonk feature table contains a ‘biotype’; we used biotype ‘protein coding’ to restrict the output of fragment positions to the nearest protein coding genes only. However, it is possible to use other ‘biotypes’ (for example, microRNA (miRNA)) for genomic annotation. We did not impose limits on the distance of fragments from a gene; however, *identgeneloc* provides the option of applying a distance limit. Further, for fragments internal to the gene, *identgeneloc* includes an option to return information on whether the fragment is located on exons, introns or intron/exon boundaries.

### Generation of fragment-based neutrophil methylomes

We previously described a novel fragment based analysis approach for RRBS data. In this method, MspI fragments (40–220 bp in size) are used as the unit of analysis. We selected the fragments having 10 or more reads at≥2 CpG sites in each individual to provide fragment-based methylomes for these individuals (F2 t10 switch in the diffmeth program of DMAP tool^12^, see Code availability 3). Data Citation 1 contains these fragment-based methylomes for the 13 RRBS libraries described in this article.

### Code availability

1. FastQC: http://www.bioinformatics.babraham.ac.uk/projects/fastqc/
2. FASTX-Toolkit: http://hannonlab.cshl.edu/fastx_toolkit/
3. We developed several programs to facilitate genome-wide methylation data analysis. Our own software programs that are described in this manuscript (e.g., *cleanadaptors*) were originally distributed as a shell archive (meth_progs_dist.shar) along with the supplementary data sets^10^. Further, we developed a comprehensive differential methylation analysis package (DMAP)^12^. The detailed documentation of DMAP tools is available for free download from Department of Biochemistry (University of Otago) website as shell archive file. Link: http://biochem.otago.ac.nz/research/databases-software/. We have described the functionality of the DMAP programs and distributed a test data set (data for six RRBS samples, three control and three diseased patients) as part of our previous publication^12^. Although the programs were developed on a MacOS X platform, the programs have been successfully compiled with gcc and run on various Linux distributions.
4. Bismark^16^: http://www.bioinformatics.babraham.ac.uk/projects/bismark/
5. TopHat^20^: https://ccb.jhu.edu/software/tophat/index.shtml
6. SeqMonk: http://www.bioinformatics.babraham.ac.uk/projects/seqmonk/

## Data Records

RRBS data, the mapped sequenced reads for RRBS data are presented in BAM format for 13 libraries in Data Citation 1 (12 samples and one technical replicate of X9012). This dataset contains an additional RRBS sample (X9017) that was not described in our primary research article. X9016 and X9017 samples are monozygotic twins (Table 1). Quality assessment, processing and alignment of the monozygotic twin pair subjects were performed using same pipeline to other subjects as described here. For each of these samples a fragment-based DNA methylomes is also presented in.txt format (contains chromosome, start, end, length of the fragment, number of CpG sites in the fragment, count of methylated and methylated CPGs and percentage methylation of the fragment). Fragment-based DNA methylomes were also included for the monozygotic twin pair subjects. In addition, a list of variably methylated fragments and genes that were described in our previous publication is presented in Excel file format in Data Citation 1. For RNA-seq data, the mapped sequenced reads are presented in BAM format for four libraries in Data Citation 2. The FPKM values for all the genes in the genome are summarised in Excel file format for all the four samples in Data Citation 2 (file name: Neutrophil_4sample_FPKMs.xls).

## Technical Validation

### Quality control-RNA integrity

To determine quality of the RRBS libraries, a bioanalyser was used. The bioanalyser traces gave an accurate description of the fragment size and any contamination (primer contamination or adaptor dimer) in the library. A high sensitivity DNA chip was used and 1 μl of the final library was run in the Agilent 2100 bioanalyser. The sequenced libraries showed no trace of contamination or degradation and the size range was within 40–220 bp as expected for RRBS (Supplementary Figs 4 and 5, and Supplementary File 1).

The integrity of the total RNA was measured by the RNA Integrity Number (RIN) algorithm; calculated by the Agilent Bioanalyzer software (using RNA nano kit from Agilent). This method determines the quality and degradation level of the RNA from the entire electrophoretic trace of the RNA sample. This generates a RIN score, with the highest RIN score being of 10. The median RIN score of the samples was 8.04 (range: 7.8 to 8.9) indicating the high integrity of total RNAs used for sequencing (Table 2 and Supplementary Figs. 6–9, and Supplementary File 1).

### RRBS raw data quality

RRBS data were initially analyzed with FastQC (see Code availability 1) and a representative summary plot is depicted in Fig. 2c. The RRBS libraries contain fragments of 40–220 bp and the sequence runs performed for the study were of 100 bp. Therefore, fragments which are shorter than 100 bp, will contain adaptor sequences at the 3′ end. Therefore, adaptors were removed using the *cleanadaptors* (see Code availability 3) program as described previously (Fig. 2b)^10^. After adaptor removal the quality was again assessed to ensure adaptor free reads. Since the Illumina platform uses sequence by synthesis chemistry to sequence the DNA molecules and as a result of accumulation of errors, the base-calling is less accurate at the 3′ end of the reads (as shown in the Fig. 2c). The low quality sequence at the end of the reads can cause misalignment events and reduce mapping efficiency. To improve the quality of the data fastx_trimmer (see Code availability 2) was used to perform hard trimming of the sequenced reads (note: when performing hard trimming on HiSeq data, rather than GAII data, an additional –Q 33 switch was needed in the command). The decision of how many base-pairs to be hard-trimmed from the 3′ end of the read was arbitrary. For each library a decision was made based after inspecting the FASTQC reports. For example, in the given example of X9015, we decided to trim 25 bp from the 3′ end of the reads (Fig. 2c). We have previously demonstrated the rationale behind this step and performed analysis of different length of hard trimming and its relationship mapping efficiency^10^. This step not only ensures improved average quality of the sequence reads used for subsequent analysis but also reduced the rate of mismatches during mapping with the reference genome which in turn improved alignment efficiencies^10^.

### RNA-Seq raw data quality

**Figure 3:**
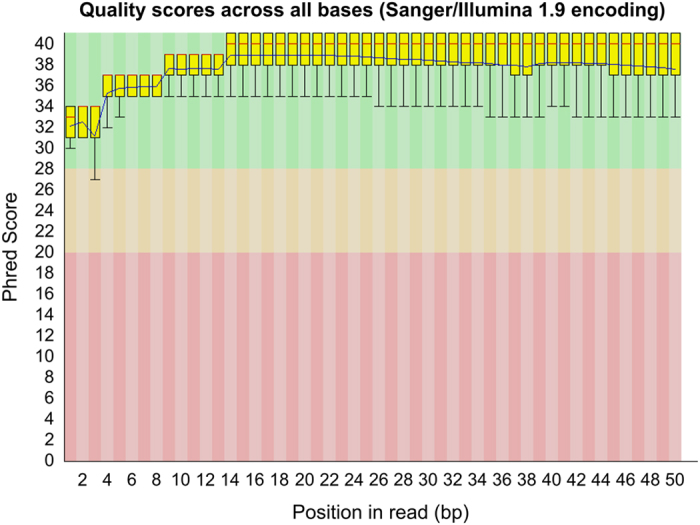
A representative example of per base sequence quality for RNA-Seq data (sample: X9015). The plot was generated using FastQC program. See Fig. 2c legend for explanation.

The quality of the raw sequenced reads from RNA-Seq experiments was excellent as indicated by FASTQC. An example of Phred score quality of sequenced reads is shown in Fig. 3 for sample X9015, and all other libraries showed similar quality metrics. The median Phred scores values was>34 till the last cycle. Therefore hard-trimming was not necessary for this data. We assessed adaptor the traces and removed them using *cleanadaptors* before mapping.

### RRBS technical replicates

We also included a technical replicate RRBS library (sample: X9012) in our study. The reason for including a technical replicate library was to assess the extent to which technical artifacts affect DNA methylation quantification. The technical replicate library was prepared with the same DNA material as X9012 (same DNA extraction) and the same TruSeq kit used for X9012. These two libraries were sequenced in different flow cells. To assess the technical reproducibility, we compared methylation of common fragments between these technical libraries using a Bland-Altman (BA) plot. We found that the 95% limits of agreement were −11.7 to 11.5% methylation demonstrating high reproducibility of RRBS data (see primary article for BA plot^8^). This observation is consistent with previous reports of reproducible results from RRBS data^24,25^. We also carried out analysis using the methylKit program^26^. For this analysis, X9012 and X9012_replicate libraries were processed in the methylKit package and only CpG sites that were covered by at least 10 sequenced reads in both the samples were included in the calculation of Pearson’s correlation coefficient (r). We observed very high positive correlation between X9012 and X9012_replicate samples (r=0.98, see Supplementary Fig. 10, Supplementary File 1). However, as methylation distribution in somatic cell is bimodal (i.e., heavily biased towards methylated or unmethylated patterns), Pearson’s correlation analysis can confound interpretation of reproducibility; thus the Bland-Altman method is more suitable for this purpose.

### Assessment of stable expression of house keeping genes

Previously, by using real-time PCR experiments, Zhang *et al.* showed that seven genes (*HPRT1, TBP, RPL32, GNB2L1, GAPDH, ACTB, B2M*) showed extremely stable expression in human neutrophils^17^. To test whether we see a stable and non-variable expression in these genes in our individuals, we plotted log_2_ of the FPKM values for the seven neutrophil housekeeping genes. We were able to reproduce Zhang’s findings and our RNA-Seq data confirmed the expression stability of these genes and minimal variation in gene expression between individuals (standard error of the mean ranged from 0.07 (RPL32) to 0.33 (B2M); see our primary paper for the relevant figure^8^).

## Usage Notes

Although genetic variation is relatively well understood, much less is known about epigenetic variation, especially in normal populations. The dataset presented here will allow further analysis and understanding of the mechanisms of sources of DNA methylation variation in healthy individuals and also investigate the relationship between genetic and epigenetic variation. Detailed understanding of inter-individual variation in normal populations is necessary from a practical clinical point of view. For the development of more confident detection of aberrant methylation in diseased patients, it is crucial to know the range of methylation that a healthy individual could exhibit. For robust DNA methylation biomarker development it is important to choose sites (CpGs), which show minimal variability in healthy individuals and significant variation between patient and controls^27,28^. Our dataset will facilitate these comparisons.

Our data provides genome-wide methylation and transcriptome profiles from a cohort of normal individuals of different ethnicity. The addition of our RRBS and RNA-Seq datasets to those of emerging genome wide studies in human blood cell types should facilitate comparisons between studies, provide valuable correlative information, and accelerate the development of online hubs to enable future comparisons of human epigenetic data. For example, the BLUEPRINT project is in the process of generating epigenomic datasets of different blood cell types from healthy and diseased individuals^29^. Our data is complementary to the BLUEPRINT data and will provide an additional resource.

During the analysis of these datasets we have developed several computational tools and pipelines and we have distributed these tools freely with a test dataset for wider use. The analysis approach and tools described here will facilitate bioinformatics analysis. The DMAP pipeline contains a suite of statistical tools and analytical approaches for large-scale DNA methylation analysis^12^. The program can analyse RRBS and whole genome bisulfite sequencing (WGBS) rapidly since it runs as compiled code of the C language. These tools will be useful for other users performing next generation sequencing data analysis.

## Additional Information

**How to cite this article:** Chatterjee, A. *et al.* Genome-scale DNA methylome and transcriptome profiling of human neutrophils. *Sci. Data* 3:160019 doi: 10.1038/sdata.2016.19 (2016).

## Supplementary Material



Supplementary Information

## Figures and Tables

**Table 1:** Details of the participants for which RRBS data was generated.

**Sample ID**	**Gender**	**Age**	**Ethnic Origin**	Processed reads (millions)*	**Length after hard trimming**
X9015	Male	26	Indian subcontinent	18.4	75
X9006	Female	31	Southeast Asia	21.7	65
X9010	Female	34	United Kingdom	30.9	75
X9007	Female	25	Indian subcontinent	9.1	100
X9019	Female	33	Eastern Europe	28.3	80
X9020	Male	34	Southeast Asia	17.9	80
X9014	Male	31	United Kingdom	18.7	90
X9012	Male	29	South America	31.3	85
X9018	Male	33	Pacific islands	43.8	85
X9016	Female	32	Western Europe	45.6	80
X9017	Female	32	Western Europe	23.6	80
X9021	Female	28	Pacific islands	43.1	80
X9012 _replicate	Male	29	South America	16.3	85

**Table 2:** Details of the participants for which RNA-Seq data was generated.

**Sample ID**	**Gender**	**Age**	**Ethnic Origin**	**RIN value**	Processed reads (millions)*
X9015	Male	26	Indian subcontinent	7.90	38.2
X9010	Female	34	United Kingdom	7.80	72.1
X9019	Female	33	Eastern Europe	8.20	44.5
X9014	Male	31	United Kingdom	8.90	19.6
