# Supplementary material for: Genome-scale DNA methylome and transcriptome profiling of human neutrophils
Source: Sci Data. 2016 Mar 15;3:160019. doi: 10.1038/sdata.2016.19 (PMC4792177; doi:10.1038/sdata.2016.19)
Supplement: Supplementary Information [file sdata201619-s2.pdf]

Supplementary File 1 for:

Genome-scale DNA methylome and transcriptome profiling of human neutrophils

Aniruddha Chatterjee[1, 2]*, Peter A. Stockwell[3], Euan J. Rodger[1], Ian M. Morison[1]*

1. Department of Pathology, Dunedin School of Medicine, University of Otago, 270 Great King Street, Dunedin 9054, New Zealand institution
2. Maurice Wilkins Centre for Molecular Biodiscovery, Level 2, 3A Symonds Street, Auckland, New Zealand
3. Department of Biochemistry, University of Otago, 710 Cumberland Street, Dunedin 9054, New Zealand

* To whom correspondence should be addressed: Aniruddha Chatterjee, Department of Pathology, Dunedin School of Medicine, University of Otago, P.O. Box 913, Dunedin, New Zealand, Telephone: +64 3 470 3455, E-mail: aniruddha.chatterjee@otago.ac.nz

Supplementary Figures S1 – S10.

Supplementary Tables S1.

Supplementary Figures

Sample No.:	JEF1	Rack:	Tube: 0	29/06/2011	14:01:54
Patient ID:		Ward:		Dr.:	
Name:				Birth:	Sex:
Comments:				Inst.ID:	SCL Dunedin

Positive
Morph. Count

WBC	3.09	-	[10^9/L]			
RBC	0.00	*	[10^12/L]			
HGB	0	-	[g/L]			
HCT	0.000	*	[Ratio]			
MCV	----		[fL]			
MCH	----		[pg]			
MCHC	----		[g/L]			
PLT	0	-	[10^9/L]			
RDW-SD	----		[fL]			
RDW-CV	----		[%]			
PDW	----		[fL]			
MPV	----		[fL]			
P-LCR	----		[%]			
PCT	----		[%]			
NEUT	2.92	*	[10^9/L]	94.5	*	[%]
LYMPH	0.08	*	[10^9/L]	2.6	*	[%]
MONO	0.00	*	[10^9/L]	0.0	*	[%]
EO	0.09	*	[10^9/L]	2.9	*	[%]
BASO	0.00		[10^9/L]	0.0		[%]
NRBC			[10^9/L]			[/100WBC]
IG	0.00		[10^9/L]	0.0		[%]
RET	----		[%]	0.0	*	[10^9/L]
IRF	----		[%]			
LFR	----		[%]			
MFR	----		[%]			
HFR	----		[%]			
RET-He	----		[pg]			
IPF	0.0	*	[%]			

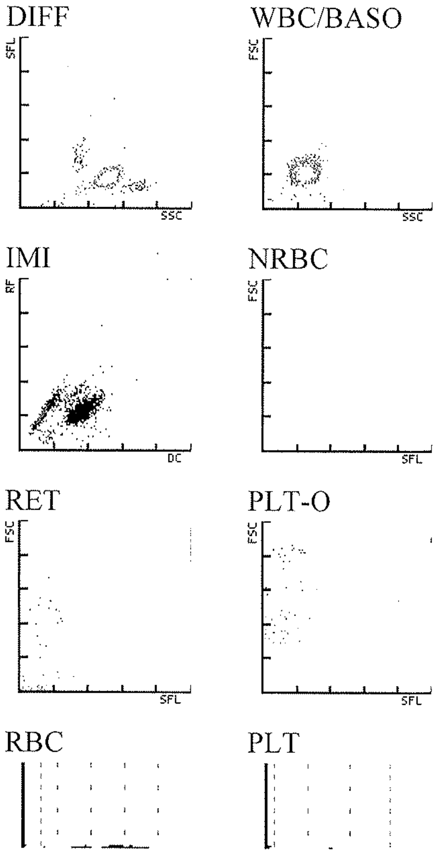

WBC IP Message(s)	RBC/RET IP Message(s)	PLT IP Message(s)
	RBC Abn Distribution	Thrombocytopenia
Blasts?		
Left Shift?		

Fig. S1: Whole blood cell count of X9010 sample before enrichment as a representative example. 200 µL of peripheral blood was used to count the cells in XE2100 Haematology Analyser. NEUT represents neutrophils. The percentage of neutrophils was 37.2% in peripheral blood before enrichment.

Sample No.:	JEF1	Rack:	Tube: 0	29/06/2011	14:01:54
Patient ID:		Ward:	Dr.:		
Name:			Birth:		Sex:
Comments:				Inst.ID:	SCL Dunedin

Positive
Morph. Count

WBC	3.09 -	[10^9/L]			
RBC	0.00 *	[10^12/L]			
HGB	0 -	[g/L]			
HCT	0.000 *	[Ratio]			
MCV	----	[fL]			
MCH	----	[pg]			
MCHC	----	[g/L]			
PLT	0 -	[10^9/L]			
RDW-SD	----	[fL]			
RDW-CV	----	[%]			
PDW	----	[fL]			
MPV	----	[fL]			
P-LCR	----	[%]			
PCT	----	[%]			
NEUT	2.92 *	[10^9/L]	94.5 *	[%]	
LYMPH	0.08 *	[10^9/L]	2.6 *	[%]	
MONO	0.00 *	[10^9/L]	0.0 *	[%]	
EO	0.09 *	[10^9/L]	2.9 *	[%]	
BASO	0.00	[10^9/L]	0.0	[%]	
NRBC		[10^9/L]		[/100WBC]	
IG	0.00	[10^9/L]	0.0	[%]	
RET	----	[%]	0.0 *	[10^9/L]	
IRF	----	[%]			
LFR	----	[%]			
MFR	----	[%]			
HFR	----	[%]			
RET-He	----	[pg]			
IPF	0.0 *	[%]			

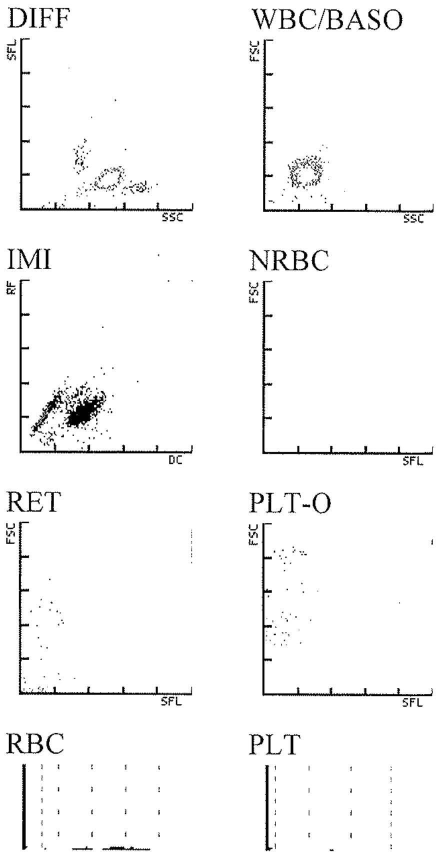

WBC IP Message(s)

Blasts?
Left Shift?

RBC/RET IP Message(s)
RBC Abn Distribution

PLT IP Message(s)
Thrombocytopenia

Fig. S2: Whole blood cell count of X9010 sample after enrichment as a representative example. 200 μL of peripheral blood was used to count the cells in XE2100 Haematology Analyser. NEUT represents neutrophils. The percentage of neutrophils was 94.2% in peripheral blood after enrichment.

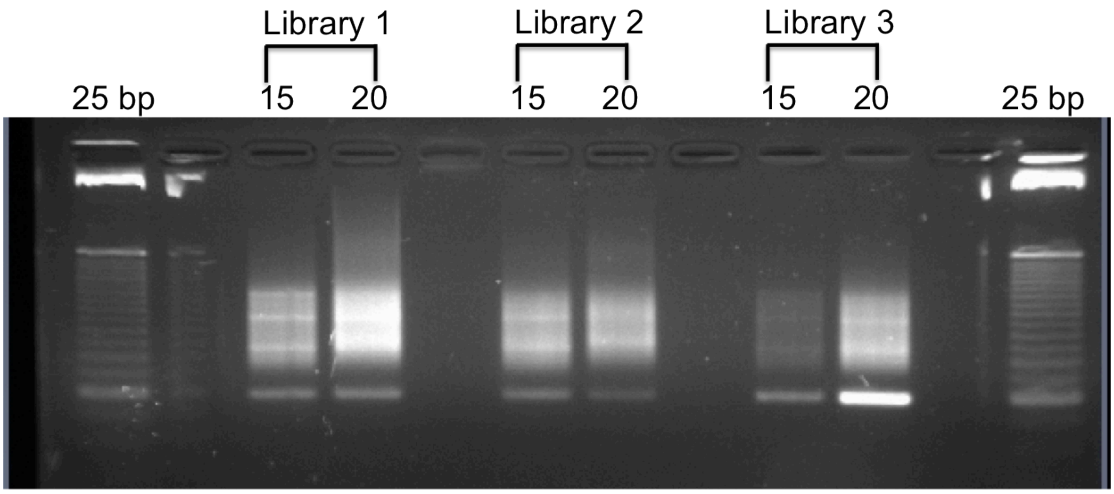

Fig. S3: Example of three successful RRBS libraries prepared following the described protocol in the manuscript. Three different RRBS libraries (X9012, X9014 and X9016) were amplified after bisulfite conversion with 15 and 20 PCR cycles and electrophoresed in 3% nusieve gel for 90 minutes (50 volts). Ladder: 25 bp (Invitrogen).

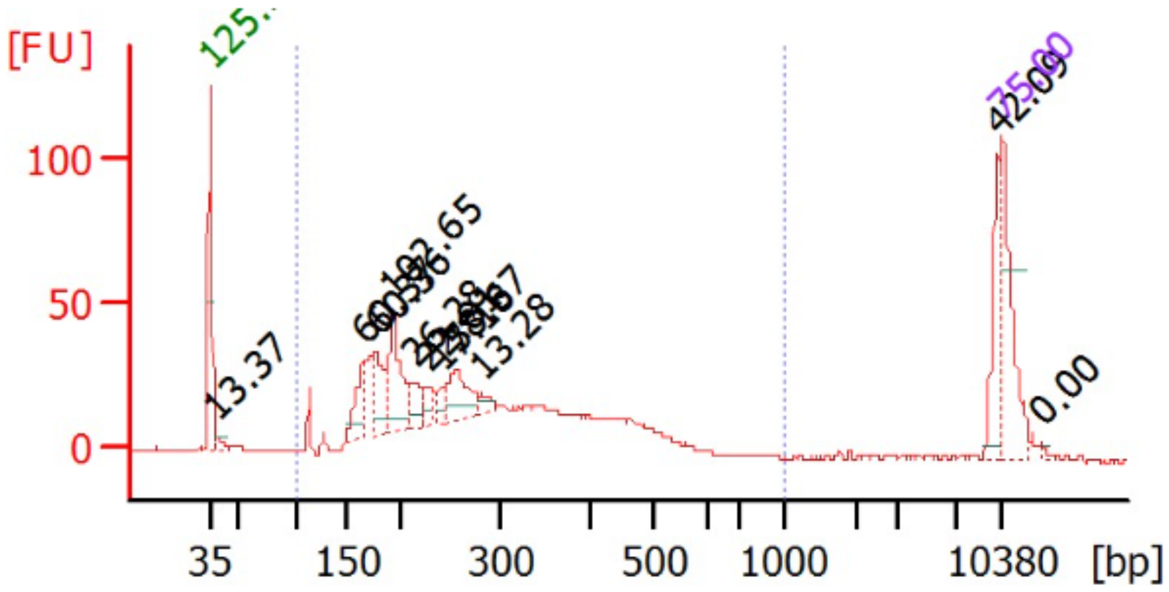

Fig. S4: A representative bioanalyzer electropherogram of a sequenced RRBS library. The prepared RRBS library was run on an Agilent 2100 Bioanalyzer using the high sensitivity DNA kit to determine the quality of the samples. The electropherogram displays a plot of fragment size in the x-axis (bp) versus fluorescence intensity (fluorescence units, FU) in y-axis. Peaks at 35 bp and 10380 bp represent lower and upper markers. The 150- 330 bp peaks represent the RRBS library (size after ligation of the adaptor, this corresponds to the actual size of 40-220 bp DNA fragments).

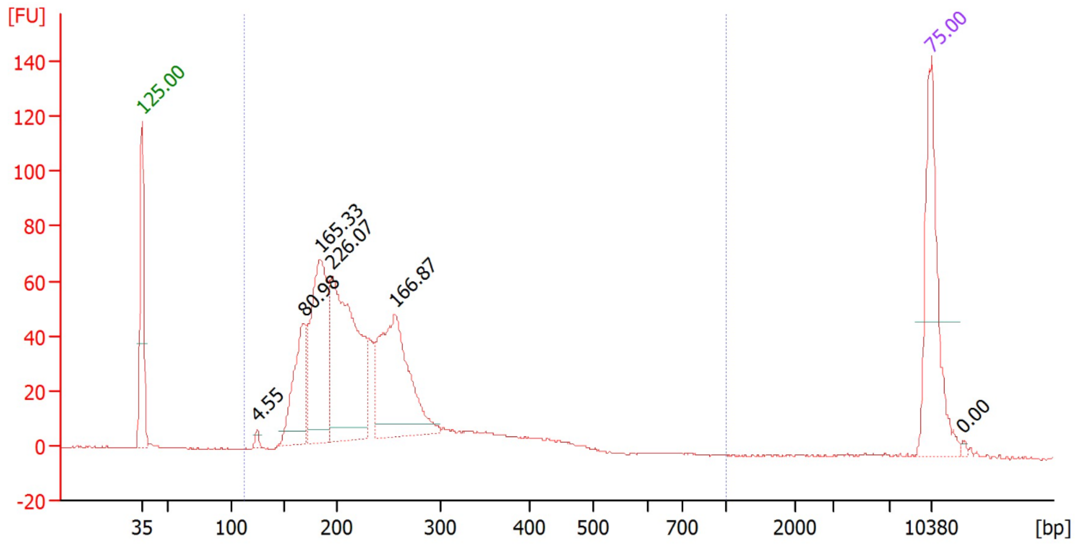

Fig. S5: A representative bioanalyzer electropherogram of a sequenced RRBS library. The prepared RRBS library was run on an Agilent 2100 Bioanalyzer using the high sensitivity DNA kit to determine the quality of the samples. The electropherogram displays a plot of fragment size in the x-axis (bp) versus fluorescence intensity (fluorescence units, FU) in y-axis. Peaks at 35 bp and 10380 bp represent lower and upper markers. The 150- 330 bp peaks represent the RRBS library (size after ligation of the adaptor, this corresponds to the actual size of 40-220 bp DNA fragments).

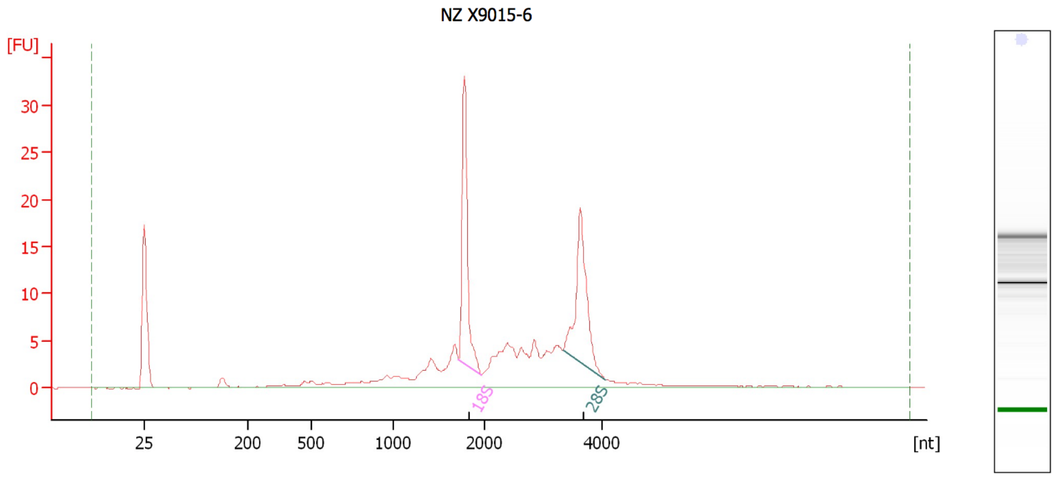

Overall Results for sample 4 : NZ X9015-6

RNA Area:	147.5	RNA Integrity Number (RIN):	7.9 (B.02.08)
RNA Concentration:	58 ng/µl	Result Flagging Color:	
rRNA Ratio [28s / 18s]:	1.0	Result Flagging Label:	RIN: 7.90

Fragment table for sample 4 : NZ X9015-6

Name	Start Size [nt]	End Size [nt]	Area	% of total Area
18S	1,716	1,960	25.7	17.4
28S	3,321	4,047	24.7	16.7

Fig. S6: Bioanalyzer electropherogram of X9015 RNA-Seq library. The prepared RRBS library was run on an Agilent 2100 Bioanalyzer using the high sensitivity RNA nano kit (from Agilent) to determine the quality of the samples. The electropherogram displays a plot of fragment size in the x-axis (nt) versus fluorescence intensity (fluorescence units, FU) in y-axis. RNA Integrity Number (RIN) is shown below in the figure. Further, RNA concentration, and ratio of different RNA submits are shown.

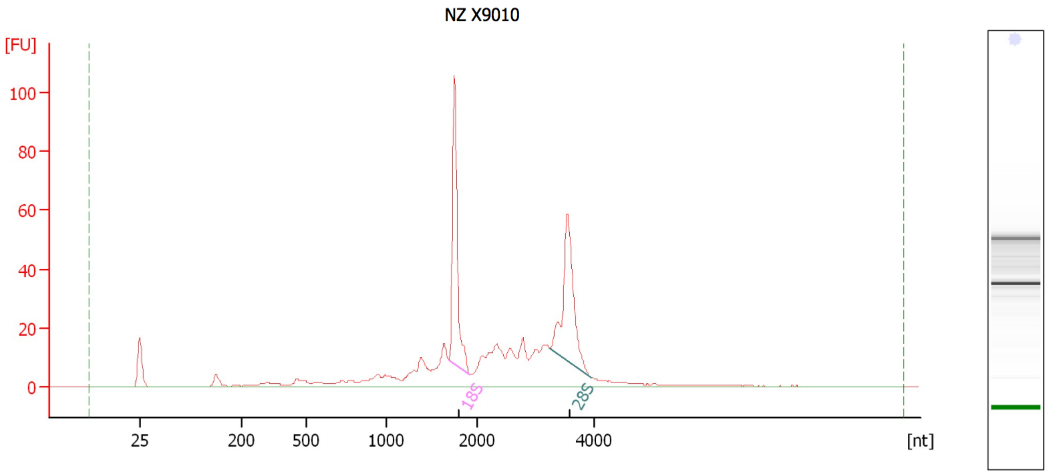

Overall Results for sample 7 : **NZ X9010**

RNA Area:	462.3	RNA Integrity Number (RIN):	7.8 (B.02.08)
RNA Concentration:	183 ng/µl	Result Flagging Color:	
rRNA Ratio [28s / 18s]:	0.9	Result Flagging Label:	RIN: 7.80

Fragment table for sample 7 : **NZ X9010**

Name	Start Size [nt]	End Size [nt]	Area	% of total Area
18S	1,686	1,922	85.0	18.4
28S	3,240	3,956	75.3	16.3

Fig. S7: Bioanalyzer electropherogram of X9010 RNA-Seq library. The prepared RRBS library was run on an Agilent 2100 Bioanalyzer using the high sensitivity RNA nano kit (from Agilent) to determine the quality of the samples. The electropherogram displays a plot of fragment size in the x-axis (nt) versus fluorescence intensity (fluorescence units, FU) in y-axis. RNA Integrity Number (RIN) is shown below in the figure. Further, RNA concentration, and ratio of different RNA submits are shown.

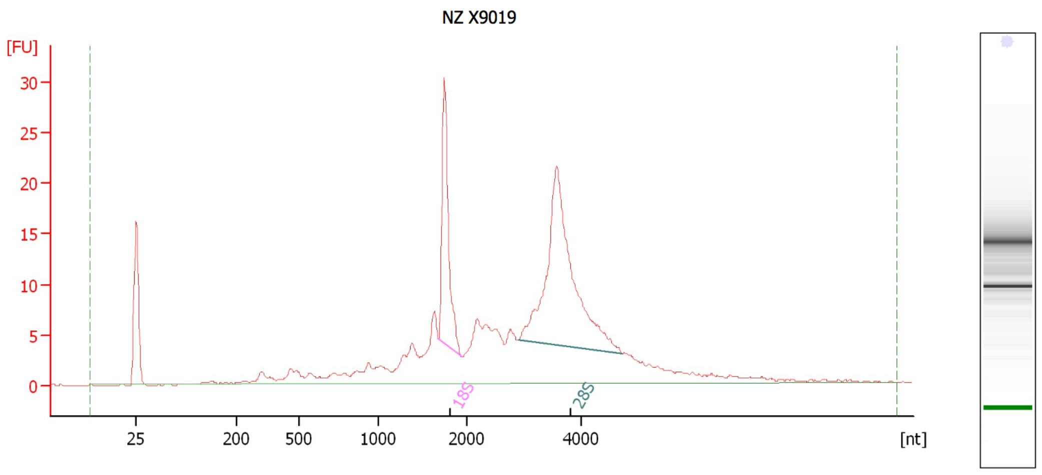

Overall Results for sample 10 : **NZ X9019**

RNA Area:	258.9	RNA Integrity Number (RIN):	8.2 (B.02.08)
RNA Concentration:	103 ng/µl	Result Flagging Color:	
rRNA Ratio [28s / 18s]:	2.4	Result Flagging Label:	RIN: 8.20

Fragment table for sample 10 : **NZ X9019**

Name	Start Size [nt]	End Size [nt]	Area	% of total Area
18S	1,678	1,944	27.9	10.8
28S	2,893	4,723	66.6	25.7

Fig. S8: Bioanalyzer electropherogram of X9019 RNA-Seq library. The prepared RRBS library was run on an Agilent 2100 Bioanalyzer using the high sensitivity RNA nano kit (from Agilent) to determine the quality of the samples. The electropherogram displays a plot of fragment size in the x-axis (nt) versus fluorescence intensity (fluorescence units, FU) in y-axis. RNA Integrity Number (RIN) is shown below in the figure. Further, RNA concentration, and ratio of different RNA submits are shown.

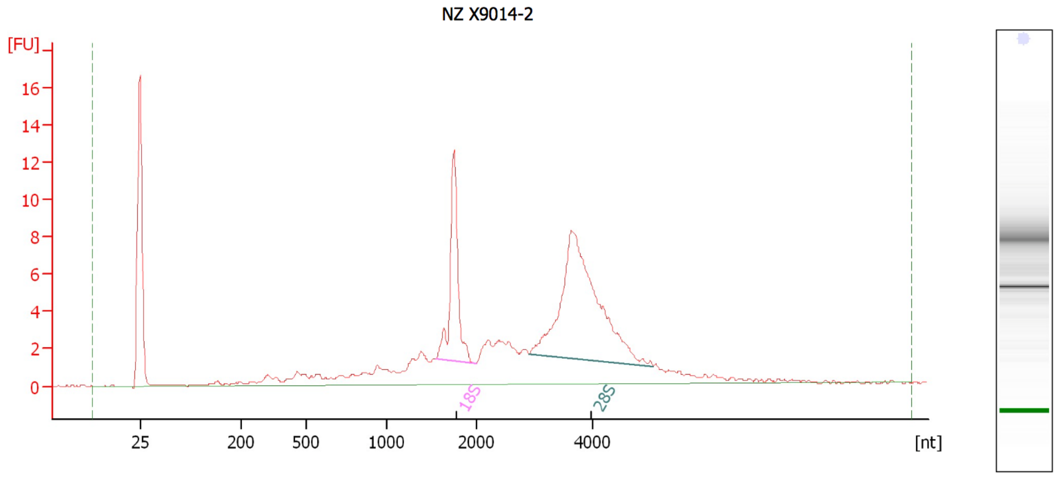

Overall Results for sample 9 : NZ X9014-2

RNA Area:	118.8	RNA Integrity Number (RIN):	8.9 (B.02.08)
RNA Concentration:	47 ng/µl	Result Flagging Color:	
rRNA Ratio [28s / 18s]:	2.5	Result Flagging Label:	RIN: 8.90

Fragment table for sample 9 : NZ X9014-2

Name	Start Size [nt]	End Size [nt]	Area	% of total Area
18S	1,552	2,001	14.3	12.0
28S	2,892	5,029	35.1	29.6

Fig. S9: Bioanalyzer electropherogram of X9014 RNA-Seq library. The prepared RRBS library was run on an Agilent 2100 Bioanalyzer using the high sensitivity RNA nano kit (from Agilent) to determine the quality of the samples. The electropherogram displays a plot of fragment size in the x-axis (nt) versus fluorescence intensity (fluorescence units, FU) in y-axis. RNA Integrity Number (RIN) is shown below in the figure. Further, RNA concentration, and ratio of different RNA submits are shown.

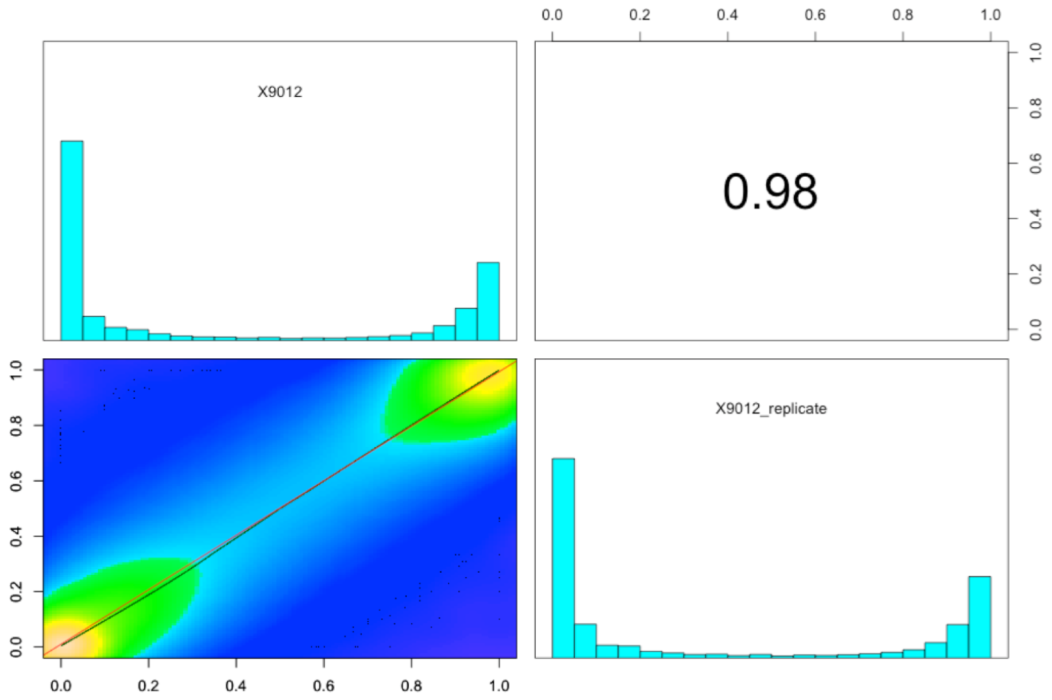

Fig. S10: Scatter plots of CpG methylation for sample X9012 and X9012_replicate. Lower left is the scatter plot of CpG methylation values for each sample. The number on upper right denotes pair-wise CpG base Pearson's correlation score for X9012 and X9012_replicate samples. The histograms show the frequency of CpG methylation in these two samples. The X and Y axis scales of the scatter plot in lower left was modified to 0.0-1.0 by methylKit program, instead of 0-100 percent methylation as shown in Figure 6.4. In this plot 0.0 represents complete unmethylation (0%) and 1.0 represents complete methylation (100%).

Supplementary Tables

Table S1. Neutrophil isolation percentages and DNA concentrations of the sequenced samples

Sample ID	Neutrophil isolation (%)	Amount of DNA (μg)	260/280 ratio on Nanodrop
X9015	96.0	38.5	1.81
X9006	96.5	27.4	1.83
X9010	94.7	43.0	1.87
X9007	97.0	26.5	1.89
X9017	94.0	49.2	1.87
X9019	96.0	34.5	1.87
X9020	97.1	40.9	1.88
X9014	92.0	20.6	1.89
X9016	96.5	66.2	1.86
X9018	90.0	50.0	1.87
X9012	95.5	68.0	1.87
X9021	97.4	50.0	1.88
